# Multi-agent cooperative swarm learning for dynamic layout optimisation of reconfigurable robotic assembly cells based on digital twin

**DOI:** 10.1007/s10845-023-02229-7

**Published:** 2024-01-17

**Authors:** Likun Wang, Zi Wang, Kevin Gumma, Alison Turner, Svetan Ratchev

**Affiliations:** Centre for Aerospace Manufacturing, University of Nottingham, Advanced Manufacturing Building, Nottingham, Nottinghamshire NG7 2GX UK

**Keywords:** Cooperative learning, Swarm optimisation, Multi-agent system, Reconfigurable manufacturing system, Digital twin

## Abstract

To meet the requirement of product variety and short production cycle, reconfigurable manufacturing system is considered as an effective solution in addressing current challenges, such as increasing customisation, high flexibility and dynamic market demand. Dynamic factory layout design and optimisation are the crucial factors in response to rapid change in the mechanical structure, software and hardware integration, as well as production capability and functionality adjustment. Nevertheless, in the current research, the layout design for reconfigurable manufacturing systems is usually simplified with autonomous devices being regarded as 2D shapes. Issues such as overlapping and transportation distance are also addressed in an approximate form. In this paper, we present a novel multi-agent cooperative swarm learning framework for dynamic layout optimisation of reconfigurable robotic assembly cells. Based on its digital twin established in the proposed learning environment (constructed in Visual Components and controlled by TWINCAT), the optimisation framework uses 3D digital representation of the facility models with minimal approximation. Moreover, instead of using a traditional centralised learning manner, multi-agent system could provide an alternative way to address the layout issues combined with the proposed decentralised multi-agent cooperative swarm learning. In order to verify the application feasibility of the learning framework, two aerospace manufacturing use cases were implemented. In the first use case, the layout compactness is reduced by 3.8 times compared with the initial layout setting, the simulated production time is reduced by 2.3 times, and the rearrangement cost decreased by 33.4$\%$. In addition, all manufacturing activity within the cell can be achieved with a feasible robot path, meaning without any joint limits, reachability or singularity issue at each key assembly point. In the second use case, we demonstrated that with the proposed dynamic layout optimisation framework, it is possible to flexibly adjust learning objectives by selecting various weight parameters among layout compactness, rearrangement cost and production time.

## Introduction

As the global manufacturing market becomes more competitive, companies are encountering notable difficulties in manufacturing high-quality products that are not only cost-effective but also have a short production lead time. Rapid changes of existing product families, large fluctuations in demand scale and production volume, and demanding responsiveness to change and manufacturing techniques bring more uncertainties into today’s market (Maganha et al., 2019). All these challenges require the investigation of a manufacturing system that could accommodate various products with short lead time and a high degree of customisation.

Reconfigurable manufacturing systems (RMS) proposed in Koren et al. (1999) are capable of dealing with the challenges mentioned above. It focuses on the rapid reconfiguration and adjustment to quickly respond to changes in product capability and functionality (Maganha & Silva, 2017). This often involves layout reconfiguration, which is the physical relocation and rearrangement of resources, such as tooling, machines, manipulators and other autonomous devices, to achieve the strategic objectives. In addition, layout reconfiguration is considered as one of the key design issues of a RMS (Hosseini-Nasab et al., 2018; Yelles-Chaouche et al., 2021). It can effectively influence the productivity levels and efficiency of production processes.

A successful layout planning would not only reasonably allocate different facilities, adequately utilise space and minimise security risk but also guarantee the production requirements to be met at a low cost (ElMaraghy, 2008). To a certain extent, the current layout could be flexibly reconfigured given additional production adjustment. In contrast, inappropriate layout might cause poor workspace utilisation, redundant workload, unacceptable production efficiency and lead time. “All this can entail anxiety and ill ease for workers, accidents at work, and make the control of operations and personnel management difficult” as outlined in Pérez-Gosende et al. (2021). For example, a loosely-packed layout can lead to extra transportation effort, increase production lead time and poor personnel arrangement.

Through the whole planning horizon, if requirements remain unchanged for manufacturing processes, the facility layout planning is considered as a static issue, which means there will be no further change required after the initial configuration. However, with demands to change and adapt, the production lines requirements can vary regularly. Therefore, it is more reasonable to consider a dynamic layout planning for each time period and different situations. In compliance with this, the number of dynamic layout planning studies is much less compared to static planning studies as pointed out in Hosseini-Nasab et al. (2018).

Although layout planning is crucial for RMSs, there are several open issues that need addressing.

Firstly, the selection of a facility layout scheme always involves asynchronous, complex and iterative production processes. The variation of these processes might leads to significant layout changes given different rating criteria. Derived from the computational complexity theory, the facility layout planning of a manufacturing system is investigated as a non-polynominal hard optimisation problem and there is no optimal solution in a reasonable polynominal time (Grobelny & Michalski, 2017).

**Fig. 1:**
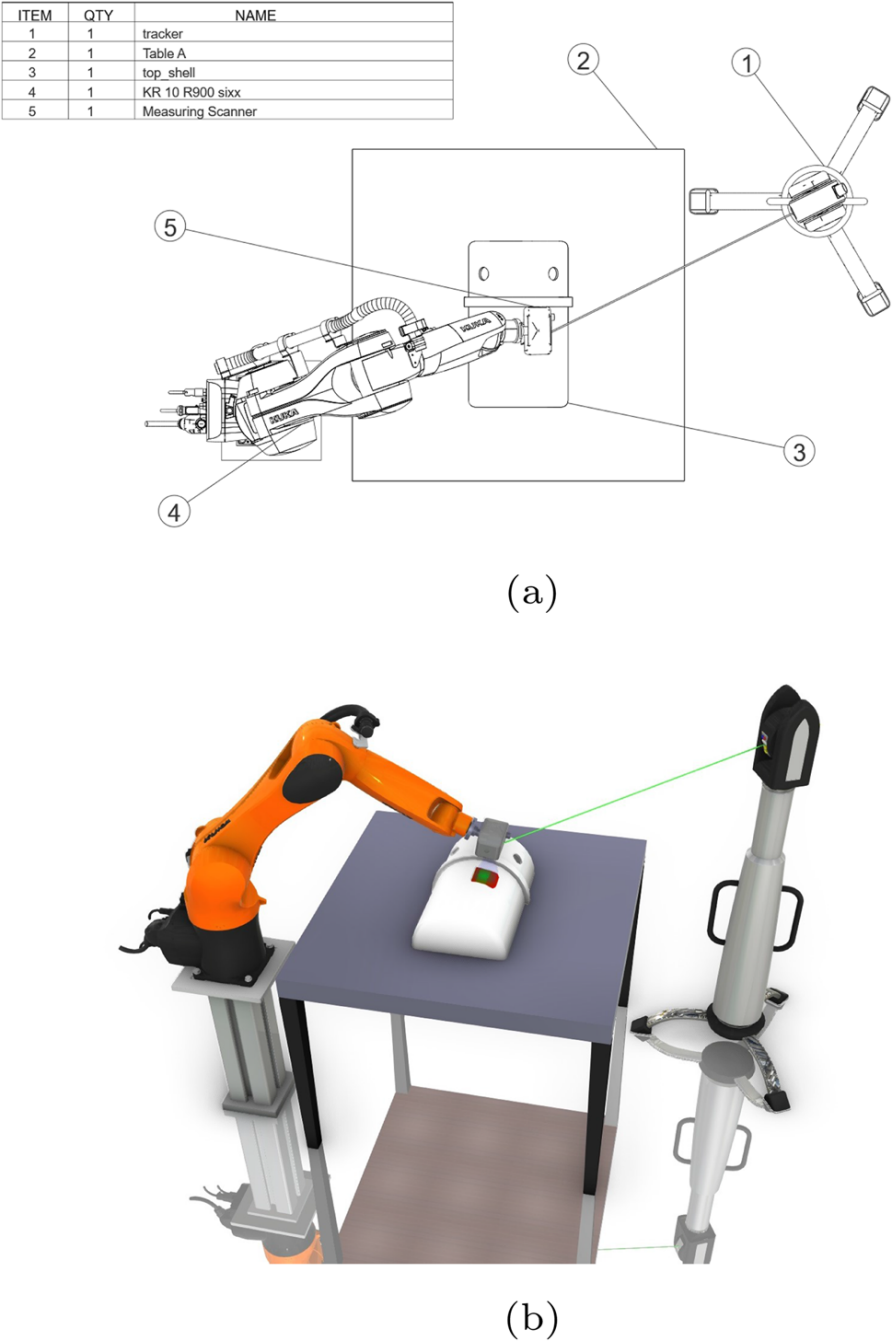
Comparison between the 2D projection model and the digital twin model in the layout optimisation. **a** Top view of the metrology cell. **b** The digital twin of the metrology cell. Compared with the top view of the metrology cell which is a 2D projection, the digital twin model could provide more comprehensive information, such as Cartesian scan trajectory, laser tracker motion and other spatial information

**Fig. 2:**
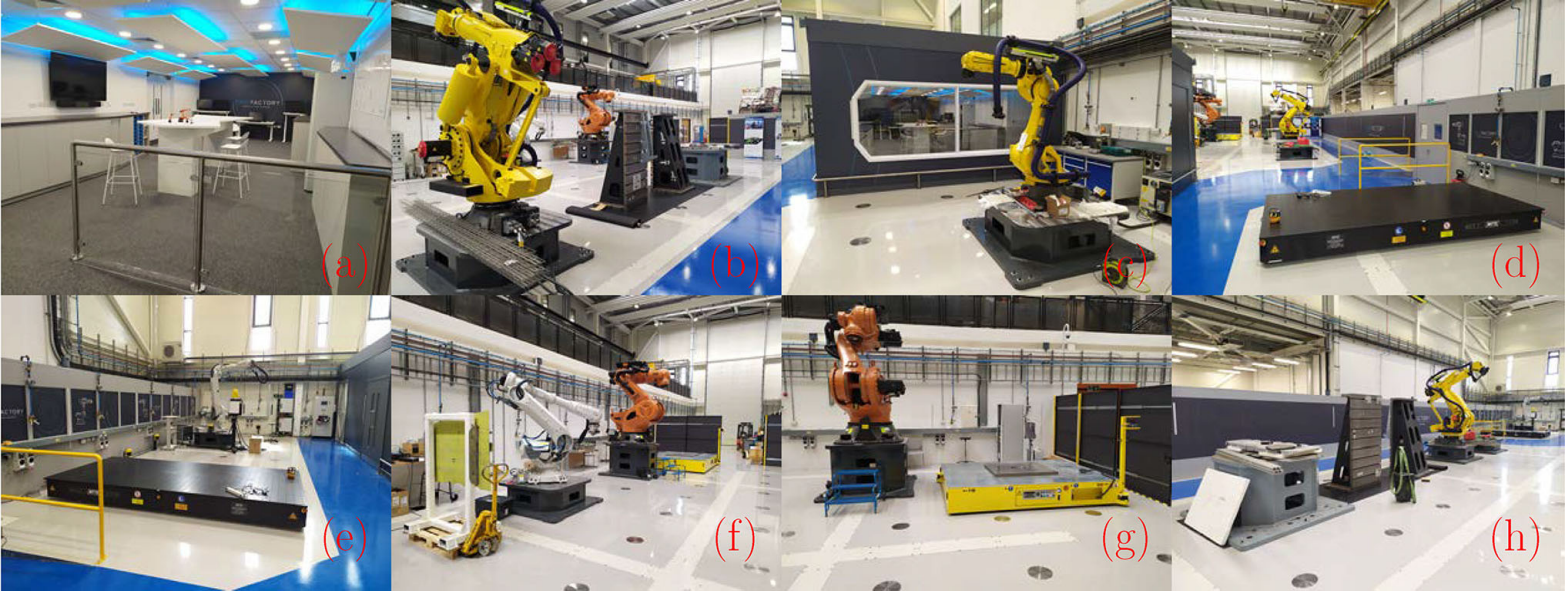
Omnifactory (Future Automated Aircraft Assembly Demonstrator Phase 2, FA3D2). The Omnifactory is designed as a experimental testbed for digital, informatics enabled, and reconfigurable aerospace manufacturing. As shown above, we present eight different views of the overall factory. For more details of this project, please refer to the official website

Secondly, almost all the facility layout planning problems in previous literatures were studied with approximated 2D facility models. Despite the minimal computational effort, the information that a 2D model provides is extremely limited. As shown in Fig. 1a, the 2D projection (top view) of a metrology work cell only provides information of the facility locations on the floor. However, if the robot workspace is constrained and the workpiece requires 3D measurements (as presented in Fig. 1b), only dimensional and positional information is not sufficient to give an overall judgement of the application feasibility (in real-world prouduction environment such as Fig. 2).

Thirdly, since most of the facility models are investigated in 2D space, the facility layout problems are limited in a certain period of production processes, a single layout criterion or an assumed situation which makes the simplified facility models or mathematical models only applicable to a certain change of the production environment. Nevertheless, for a dynamic production layout or under a complex manufacturing assembly environment, these kinds of layout optimisation results given by 2D models are usually undesirable.

In this paper, layout optimisation of reconfigurable robotic assembly cells is explored based on their digital twin in station level and machine level. The advantages include accurate representation of digital functions and mechanical features of the physical manufacturing system, full lifecycle horizon support, and data accessibility and learning feasibility both in machine level and station level.

Moreover, besides the traditional optimisation objectives, namely material handling cost and rearrangement cost, there are indeed additional objectives that are included in this work, which are collision detection, and target reachability and manipulability. They are of great importance in any robotic process. If the objective of minimising material handling cost is to generate a compact layout, the aim of collision detection is to avoid a so compact layout that facilities overlap with each other. Additionally, any robot path and target point should be checked for reachability and manipulability (to avoid joint singularity). Hence they are also optimised in this work.

Finally, unlike traditional centralised control approaches, multi-agent system consists of intelligent agents that would interact to achieve collective goals. Its modularity, flexibility and reconfigurability naturally coincide with the fundamental principle of a reconfigurable robotic assembly cell, where resources in a work cell are considered as agents. Each agent should select the positions according to their own interests calculated by different objectives. Therefore, the issue facing in this paper is a multi-objective multi-agent dynamic facility layout of reconfigurable robotic assembly cells. In order to address this issue, a bio-inspired cooperative swarm learning framework is proposed. In this learning framework, the components in the work cell are allowed to relocate and purse their own interests both from itself and the others. For each searching episode, the learning framework combines agent local views and updates the layout of reconfigurable robotic assembly cells given the multi-objective exploration results.

The main contributions of this paper are highlighted as follows,

1. Firstly, a novel digital-twin learning environment based on Visual Components and TWINCAT is established;
2. A multi-agent learning scheme for the layout optimisation of reconfigurable robotic assembly cells is introduced in this paper;
3. In addition, a cooperative swarm learning framework is proposed to find the optimal layout solution for the multi-objective multi-agent robotic assembly cell in both station level and machine level;
4. Finally, two use cases are conducted to demonstrate the application feasibility of the proposed layout optimisation methodology.

The remainder of the paper is organised as follows: after the literature review given in Literature review, the multi-agent reconfigurable robotic assembly cell is presented in Multi-agent systems for layout reconfiguration of robotic assembly cells, followed by the cooperative swarm optimisation methodology, as well as the novel digital-twin based learning environment, introduced in Cooperative interaction in addition, the application feasibility of the proposed framework is verified with two use cases in Evaluation; finally, the discussion is given in Discussion and the conclusion is drawn in Conclusion.

## Literature review

The literature review is organised in three parts. Firstly, the multi-agent system for layout reconfiguration is investigated in Multi-agent system. In addition, the digital twin for manufacturing is reviewed in Static and dynamic layout optimisation. Finally, related studies for static and dynamic layout optimisation are given in Static and dynamic layout optimisation.

### Multi-agent system

Compared with traditional centralised control approach, multi-agent system offers effective solutions, especially for the manufacturing systems that require rapid reconfiguration (Leitão et al. 2012, Kim et al., 2020). Current research of multi-agent system for industrial applications focuses more on parallel machine scheduling (Owliya et al., 2012; Barenji et al. 2017; Zhang & Wong, 2017; Maoudj et al., 2019), negotiation among distributed agents regarding production scheduling (Huang & Liao, 2012), and negotiation protocol of a smart factory framework (Wang et al. 2016).

**Table 1:** An overview of the static facility layout planning problems

References	Objectives	Levels	Models	Layout generation approaches
Benderbal and Benyoucef (2019)	Constraint penalty	Factory	2D	Mathematical modelling
Eguia et al. (2017)	Transportation costs	Machine	2D	Mathematical modelling
Che et al. (2017)	Material handling costs	Factory	2D	Mathematical modelling
Liu et al. (2021)	Material handling costs	Factory	2D	Mathematical modelling
García-Hernández et al. (2020)	Material handling and transportation cost	Station	2D	Mathematical modelling
García-Hernández et al. (2020)	Material handling costs	Station	2D	Mathematical modelling
Dahlbeck (2021)	Non-overlapping and Material handling costs	Factory	2D	Mathematical modelling
Palubeckis (2017)	Products flow costs	Factory	2D	Mathematical modelling
Sharma and Singhal (2017)	Factors for evaluation	Factory	2D	Mathematical modelling
Zhou et al. (2017)	Material handling costs	Factory	2D	Mathematical modelling
Asef-Vaziri and Kazemi (2018)	Non-vehicle-based material handling costs	Factory	2D	Mathematical modelling
Besbes et al. (2021)	Material handling costs	Factory	3D	Mathematical modelling
Azimi and Soofi (2017)	Material handling costs complication time of jobs	Factory	2D	Computer-aided planning tools
Defersha and Hodiya (2017)	Transportation costs	Factory	2D	Mathematical modelling
García-Hernández et al. (2019)	Material handling efficiency	Station	2D	Mathematical modelling
Grobelny and Michalski (2017)	Material handling efficiency	Station	2D	Mathematical modelling
Kang and Chae (2017)	Material handling efficiency	Station	2D	Mathematical modelling
Kim and Chae (2021)	Material handling costs	Station	2D	Mathematical modelling
Ning and Li (2018)	Row interaction costs	Station	2D	Mathematical modelling
Palomo-Romero et al. (2017)	Unequal area layout	Factory	2D	Mathematical modelling
Safarzadeh and Koosha (2017)	Material handling costs	Factory	2D	Mathematical modelling
Liu et al. (2020)	Material handling costs closeness rating	Station	2D	Mathematical modelling
Xie et al. (2018)	Material handling costs	Station	2D	Mathematical modelling
Park et al. (2018)	Safety considerations	Station	2D	Mathematical modelling
Feng et al. (2018)	Material handling efficiency	Station	2D	Mathematical modelling
Allahyari and Azab (2018)	Material handling efficiency	Station	2D	Mathematical modelling
Ahumada et al. (2018)	Risk assessment	Station	2D	Mathematical modelling
Durmusoglu (2018)	Closeness requirement	Factory	2D	Mathematical modelling
Ejeh et al. (2018)	Optimal spatial arrangement	Factory	3D	Mathematical modelling
Feng and Che (2018)	Total material flow	Factory	2D	Mathematical modelling
Friedrich et al. (2018)	Total material flow	Factory	2D	Mathematical modelling
Wan et al. (2022)	Material flow costs layout area	Factory	2D	Mathematical modelling
Kalita and Datta (2018)	Material handling costs	Factory	2D	Mathematical modelling
Kang et al. (2018)	Material handling costs	Factory	2D	Mathematical modelling
de Lira-Flores et al. (2019)	Land and pipeline costs	Station	2D	Mathematical modelling
Abdollahi et al. (2019)	Quality and quantity	Factory	2D	Mathematical modelling
Singh and Ingole (2019)	Quality and quantity	Factory	2D	Mathematical modelling
Liu and Liu (2019)	Material handling cost closeness rating	Factory	2D	Mathematical modelling

However, the number of studies that address RMS layout planning based on multi-agent systems is limited, within which 2D projection was used. In Tarkesh et al. (2009), a multi-agent system was applied in layout optimisation based on the fuzzy theory for establishing each agent’s utility function. However, as pointed out in Tarkesh et al. (2009), “the approach presented here has certain disadvantages in dealing with other facility layout design objectives such as department shape and total layout plant shape”. In Chraibi et al. (2014), the design of operating theatre layout was discussed with multi-agent systems by using mixed integer linear programming.

Moreover, multi-agent systems were applied in urban area layout planning as introduced in Zhang et al. (2019). In Huang et al. (2020), the rural settlement issues were addressed by combining system dynamics models and evaluation modules derived from multi-agent system. In addition, mult-agent systems were applied for indoor furniture layout optimisation (Di & Yu, 2021).

In summary, although multi-agent systems have been applied for manufacturing systems in terms of resource scheduling, negotiation and communication. However, only a few publications that addressed layout optimisation using multi-agent systems were found. As pointed out in Tarkesh et al. (2009), the main limitation is that these works used approximated facility models such as 2D projections to optimise the corresponding facility layout design. However, in a manufacturing scenario where mobile resources interact with each other in a 3D space and collision is strictly forbidden, the assumption of 2D projection quickly becomes insufficient.

### Digital twin for manufacturing

Compared with simplified bi-dimensional and tri-dimensional models, as pointed out in Xia et al. (2021), a digital twin of manufacturing system can provide high fidelity models for prediction, maintenance and monitoring. Consequently, they have been used in manufacturing for process design (Zhang et al., 2020), shop-floor system design (Li et al., 2021), and composite assembly (Polini & Corrado, 2020).

Regarding layout optimisation, in Guo et al. (2021), the discrete manufacturing workshop was optimised by using digital-twin data and physical interaction fusion. Moreover, in Nåfors et al. (2020), the virtual reality derived from the digital twin model was used to visualise facility layout and improve solution fidelity at an early stage. Furthermore, automatic layout configuration of a production line for robot positioning was proposed in Braun et al. (2021).

Additionally, a digital twin for fixed-position assembly islands was introduced in Guo et al. (2020). Nevertheless, this work mainly focused on resource allocation not layout optimisation. In Kousi et al. (2021), real-time data was used to align the positions for 3D models in a digital twin virtual world for human-robot collaboration. In Guo et al. (2021), a flexible cellular production line was optimised based on digital twin models, where attention was focused on production layout, production scheduling and logistics. In Peron et al. (2020), the emerging technologies such as 3D scanning, indoor positioning system, motion capture system, and immersive reality are used for dynamic layout planning to reduce cost, error rate and time efforts.

In conclusion, a considerable amount of literature has been published for digital-twin application in product and process optimisation and resource allocation, and a digital-twin approach towards facility layout optimisation is still not fully studied, especially at machine and station level. For instance, factory-level layout optimisation of a discrete workshop (Guo et al., 2021) and a flexible cellular line (Guo et al., 2021) were investigated, where the distances among the 2D projections of the different digital twin models were optimised and the interaction among different devices and stations were ignored.

**Table 2:** An overview of the dynamic (RMS) facility layout planning problems

References	Objectives	Levels	Models	Layout generation approaches
Azevedo et al. (2017)	Material handling and rearrangement costs	Factory	2D	Mathematical modelling
Liu et al. (2017)	Material handling and rearrangement costs	Station	2D	Mathematical modelling
Kumar and Singh (2017)	Material handling costs	Station	2D	Mathematical modelling
Tayal and Singh (2018)	Material flow costs and manufacturing time	Station	2D	Mathematical modelling
Pourvaziri and Pierreval (2017)	Material handling costs and rearrangement costs	Station	2D	Mathematical modelling
Wei et al. (2019)	Material handling costs and rearrangement costs	Factory	2D	Mathematical modelling
Haddou-Benderbal et al. (2017)	Layout evaluation effort and performance metrics	Factory	–	Mathematical modelling
Derakhshan Asl and Wong (2017)	Material handling costs and rearrangement costs	Factory	2D	Mathematical modelling
Pournaderi et al. (2019)	Equipment transport and handling costs	Factory	2D	Mathematical modelling
Vitayasak and Pongcharoen (2018)	Material handling costs	Factory	2D	Mathematical modelling
Turanoğlu and Akkaya (2018)	Material handling costs	Factory	2D	Mathematical modelling
Peng et al. (2018)	Material handling costs	Factory	2D	Mathematical modelling
Li et al. (2018)	Safety, sustainability efficiency	Factory	2D	Mathematical modelling
Kulturel-Konak (2019)	Material handling costs and rearrangement cost	Factory	2D	Mathematical modelling
Xiao et al. (2016)	Material handling costs	Factory	2D	Mathematical modelling
Moslemipour et al. (2017)	Material handling costs	Factory	2D	Mathematical modelling
Ghadirpour et al. (2020)	Material handling costs	Factory	2D	Mathematical modelling
Tayal et al. (2020)	Material handling costs	Factory	2D	Mathematical modelling
Khajemahalle et al. (2021)	Material handling costs	Factory	2D	Mathematical modelling
Erik and Kuvvetli (2021)	Material handling costs	Factory	2D	Mathematical modelling
Guo et al. (2021)	Production capacity Work in progress	Factory	3D	Digital twin
Peron et al. (2020)	Time efforts error rates and costs	Factory	3D	3D mapping Immersive Reality

**Fig. 3:**
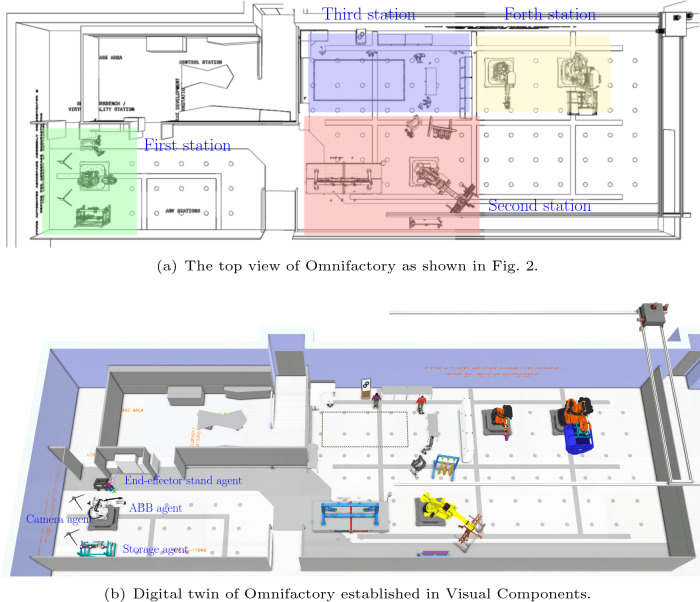
Top view and digital twin modelling of the Omnifactory. As presented in **a**, the entire factory is consisted of four robotic assembly cells (stations). In addition, the digital twin modelling is shown in **b**. Based on multi-agent system, each device is considered as a single agent. As indicated in the first station in **b**, there are five agents in the cell, i.e., two V-STARS camera agents, ABB robot agent, storage agent and end-effector stand agent

**Fig. 4:**
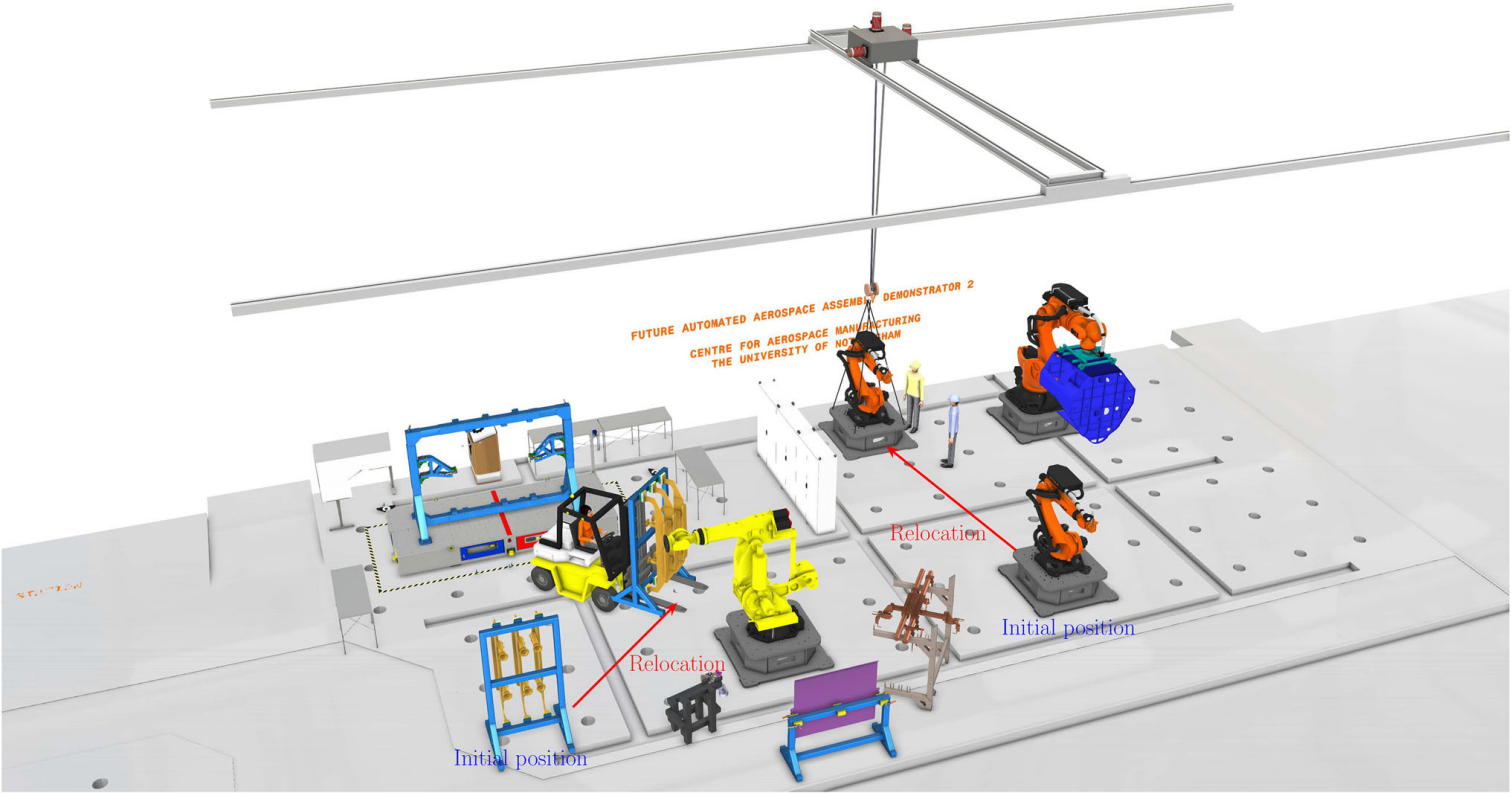
Agent relocation for layout reconfiguration. In Omnifactory, there are two main ways to relocate agents. First, agents can be relocated by crane. Second, some small devices can be moved by forklift. Note that, we design reconfigurable grids to position robot and provide pneumatic port and power supply

### Static and dynamic layout optimisation

Generally speaking, facility layout optimisation can be either considered as a static or a dynamic problem (Pérez-Gosende et al., 2021). Static layout planning (Benderbal & Benyoucef, 2019; Azevedo et al., 2017; Liu et al., 2021; Feng & Che, 2018; Friedrich, 2018) aims to solve scenarios where the material flow remain constant during the whole planning horizon among different stations. In contrast, when the layout is required to adapt to varying material flow intensity, the layout is designed to be dynamic (Pournaderi et al., 2019; Kulturel-Konak, 2019; Derakhshan Asl & Wong 2017; Li et al., 2018; 2017).

Relevant studies on static and dynamic layout optimisation are summarised in Tables 1 and 2 respectively. The general approach to layout optimisation utilises a bi-dimensional space, which is the land area of facilities. During layout optimisation, resources are approximated as rectangles (Azevedo et al., 2017; Wei et al., 2019; McKendall Jr & Hakobyan, 2010) or simplified mathematical models (Eguia et al., 2017; Haddou-Benderbal et al., 2017; McKendallJr & Shang, 2006) and their computational complexity can be reduced. In order to improve the accuracy of modelling, a number of papers focus on irregular shapes of facilities in the layout optimisation, as presented in Liu and Liu (2019), de Lira-Flores et al. (2019), Abdollahi et al. (2019), Kalita and Datta (2018).

However, it is important to note that not all layout planning can be considered as bi-dimensional. For example, a common use case of robot gantry system features robots located above the entire production line. However, only a few consider the problem in tri-dimensional space (Besbes, Zolghadri, Costa Affonso, Masmoudi, and Haddar, 2021; Yamada, Ookoudo, and Komura, 2003). In Peron et al. (2020), 3D mapping and immersive reality techniques were applied to optimise layout at a factory level to reduce the time efforts, error rates and costs. In Guo et al. (2021), facility layout was optimised regarding production capacity and work in progress using a factory digital twin. Even though a digital twin is applied, only 3D static virtual models are utilised in the layout optimisation. Machine-level and station-level interactions between resources is not considered in their work.

In summary, most of the available literature addressed static and dynamic facility layout optimisation using 2D mathematical modelling. Although a few publications investigated facility layout issues using the digital twin approach, they only focused on the factory level, where interaction between resources at machine level and station level were not captured.

## Multi-agent systems for layout reconfiguration of robotic assembly cells

In this section, the multi-agent systems for layout reconfiguration are given in threefold. Firstly, the motivation of applying multi-agent systems for layout optimisation is explained in Motivation. In addition, the agent properties including agent communication, agent state, and agent action are given in Agent state, action and communication. Finally, the agent behaviour is detailed in Agent behaviours and corresponding rewards.

### Motivation

“A multi-agent system is a system composed of multiple interacting intelligent agents that interact to solve problems that are beyond the individual capabilities or knowledge of each individual” (Weiss, 1999). In a multi-agent system, the agents should have the following three crucial characteristics (Wooldridge, 2009; Panait & Luke, 2005), i.e., autonomy, local views, and decentralisation.

Initially, autonomy in a multi-agent system defines the basic behavioural principle of an agent, which should be autonomous and partially independent. Compared this with a manufacturing system as shown in Fig. 3a, the factory consists of four cells, where various autonomous devices are required to implement production tasks. In the first station, the devices including the ABB robot, the tool storage, the end-effector stand and two V-STARS camera should autonomously complete their own individual production processes as given in Fig. 3b. Each autonomous device is independent to the other devices located at different work cells.

The second important characteristic of a multi-agent system is that each agent only has a local view of the overall system. With regard to layout reconfiguration, the optimisation objectives are based on the sum of fitness function of each agent. Hence, the agent contribution for the layout optimisation can only be obtained from their own interaction surrounding, which is local and partial.

Thirdly, in the layout reconfiguration, given that the behaviour of each agent is controlled at different operational stages during task implementation, fitness functions for optimisation are computed at different timestamps. Hence, the whole layout reconfiguration in this work is decentralised, which fulfils the third characteristic, decentralisation.

**Table 3:** Multi-agent system for layout reconfiguration

Property	Implementation
Agent commun	Event-triggered
Agent state	Position $\varvec{\xi }_n$
Agent action	Relocation
Agent behaviour	Self-interest & cooperative
Agent reward	Fitness functions

Therefore, as the layout reconfiguration naturally coincides with the three important characteristics of multi-agent systems, this paper propose a novel approach to address dynamic layout optimisation issues based on the multi-agent systems and define the following crucial properties, i.e., agent communication, agent state, agent action, agent behaviour and agent reward, as shown in Table 3. Accordingly, the first three properties are detailed in Agent state, action and communication, while the agent behaviours and their rewards are investigated in Agent behaviours and corresponding rewards.

### Agent state, action and communication

The agent state, action and communication are introduced in this subsection, respectively.

**Agent state:** In the multi-agent system for dynamic layout optimisation, agents (devices) are relocated in order to meet the requirements. Thus, each agent state is defined as the its position

1 $$\begin{aligned} \varvec{\xi }_n = \{ \xi ^x_n, \xi ^y_n, \xi ^\alpha _n \}, \end{aligned}$$

where $\xi ^x_n$ and $\xi ^y_n$ are the coordinates in the assembly cell of the *n*-th agent, and $\xi ^\alpha _n$ is the rotation angle as given in Fig. 4. For a new reconfiguration, each agent reward is only related to the agent positions.

**Agent action:** The action for a single agent is to relocate during the facility layout optimisation. As given in Fig. 4, for example, the relocation of the profile board storage is completed bu using forklift. In terms of the relocation of the robots, we design reconfigurable grids to provide power supply and pneumatic ports. For different robots, they could sit on four grids. Therefore, the relocation of a KUKA robot, for instance, is achieved with the crane as shown in Fig. 4.

**Fig. 5:**
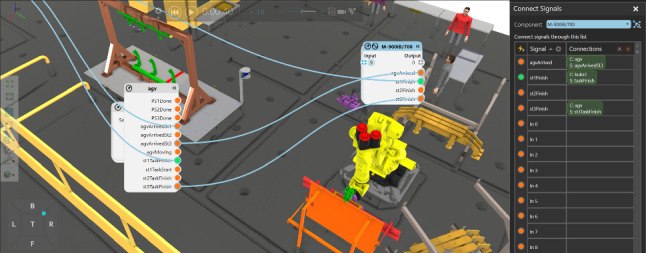
Communication among different components or agents (internal communication). The internal communication among different components is based on event-triggered communication. The signals are shown in the above figures with light blue solid lines

**Agent communication:** The communication among different agents is event-triggered and most of the signals are replicated from the Siemens PLC system in the physical robotic assembly cells, which are used for virtual commissioning. As presented in Fig. 5, the signals of the FANUC M-900iB components were divided into input signals and output signals. In addition, it can be observed that in the Connect Signals menu as shown in the right part in Fig. 5, the communication between FANUC robot, KUKA robot and AGV were realised through boolean signals. These signals were used to indicate arrival of an AGV, and the status of the first and the second station, respectively. As illustrated in Fig. 5, the status of the first task is active. In our scenario, the production process in the first station has been completed and the AGV is moving towards the second station (manual assembly).

### Agent behaviours and corresponding rewards

The agent behaviours can be divided into two categories, i.e., self-interest and cooperation. For different behaviours, there will be corresponding rewards. The self-interest behaviours are given in Self-interest, while the cooperation behaviours are presented in Cooperative interaction.

#### Self-interest

The self-interest behaviour allows agents to pursue their interests, such as AGVs and robots. In the following, several self-interest behaviours are defined along with the corresponding fitness functions (rewards).

**Facility rearrangement**: Facility layout reconfiguration is always related with facilities location rearrangement. Given the initial position $\varvec{\xi }_{n/0} = \{ \xi _{n/0}^x, \xi _{n/0}^y, \xi _{n/0}^\alpha \}$ of the agent located in the assembly cell, the rearrangement cost should be taken into account and the corresponding fitness function can be defined as

2 $$\begin{aligned} f_{arr}(\varvec{\xi }_n) = \sum _v W_{arr}(\xi _n^v - \xi _{n/0}^v)^2 , \end{aligned}$$

where $W_{arr}$ is the constant parameter for the rearrangement fitness function and the superscript $v = \{ x, y, \alpha \}$ defines the three coordinate components (two translations and one rotation around *z* axis).

**Fig. 6:**
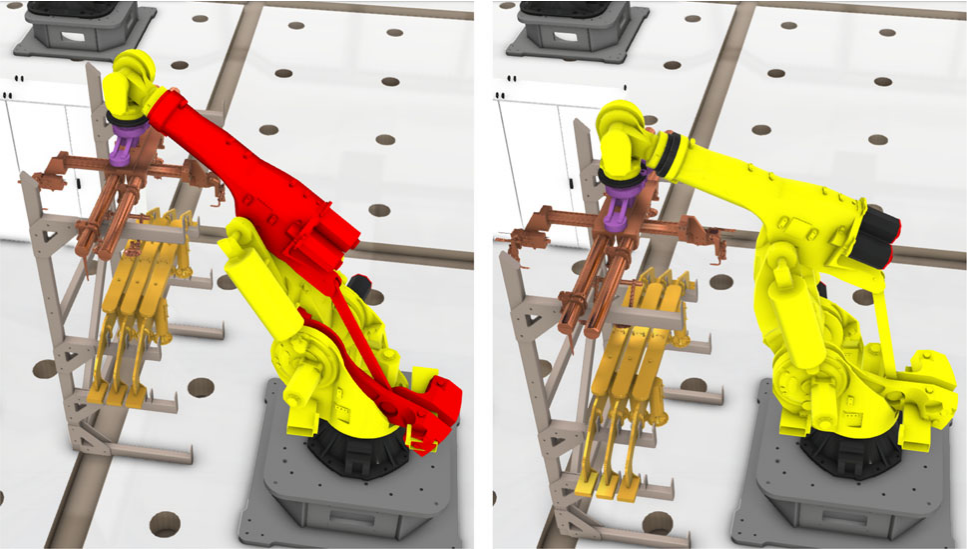
Joint limit triggered by relocation. In the left subfigure above, the robot third joint is out of the joint range. In contrast, for a different storage position in the right subfigure above, the robot third joint is in the joint range

**Robot joint limits**: In advanced robotic assembly cells, robots usually play an important role. During trajectory implementation, the robot agent should operate within its joint limits. However, due to agent relocation, the joint angle might be out of its range as indicated in red color in Fig. 6. Thus, it is necessary to taken robot joint limits into consideration in the layout optimisation. By using inverse kinematics, the target position in the Cartesian robot base frame is transformed into a joint space

$$\begin{aligned} {[}q_1, ..., q_J ] =&g_{inv}(\textsf{x}, \textsf{y}, \textsf{z}, \alpha , \beta , \gamma ) \\ \text {s.t.} \quad&q_{jn}^l \le q_j \le q_{jn}^u,\ j = 1, ..., J. \end{aligned}$$

where $g_{inv}$ is the inverse kinematic solution of the robot given a Cartesian target $(\textsf{x}, \textsf{y}, \textsf{z}, \beta _{\textsf{x}}, \beta _{\textsf{y}}, \beta _{\textsf{z}} )$, with $(\textsf{x}, \textsf{y}, \textsf{z})$ the target translations and $( \beta _{\textsf{x}}, \beta _{\textsf{y}}, \beta _{\textsf{z}} )$ the target rotations, respectively. The joint angle $q_j$ should satisfy the constraints shown in the above equation, as $q_{jn}^l$ and $q_{jn}^u$ are the lower bound and upper bound of the robot joint, respectively. Hence the fitness function can be expressed as

3 $$\begin{aligned}{} & {} f_{inv}(\varvec{\xi }_n) = \nonumber \\{} & {} \quad {\left\{ \begin{array}{ll} 0 &{} \text {if}\ q_{jn}^l \le q_j \le q_{jn}^u \\ \sum _j \Vert q_j - q_{jn}^l \Vert + \Vert q_j - q_{jn}^u \Vert &{} \text {otherwise}. \end{array}\right. } \end{aligned}$$

**Fig. 7:**
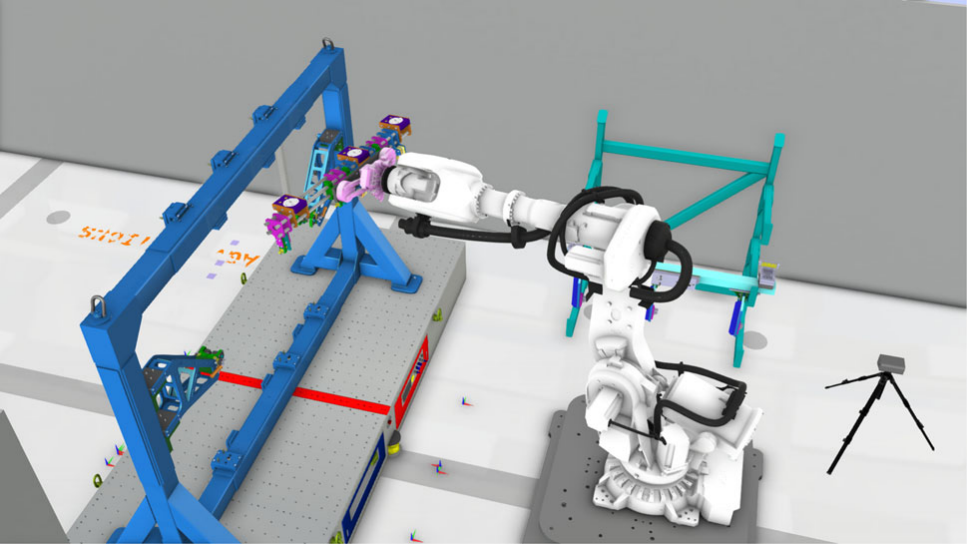
Wrist singularity triggered by relocation. The relocation might cause robot wrist singularity. Therefore, it is considered in this work and a fitness function is introduced to avoid this issue

**Table 4:** Learning objectives for the dynamic layout optimisation of the robot assembly cell

Interaction	Description	Fit. function	Objective
Self-interest	Rearrangement (Eq. 2)	$f_{arr}(\varvec{\xi }_n)$	Reduce facility rearrangement cost
	Robot joint limits (Eq. 3)	$f_{inv}(\varvec{\xi }_n)$	Check task feasibility
	Robot singularity (Eq. 4)	$f_{man}(\varvec{\xi }_n)$	Check task feasibility
Cooperative	Collision detection (Eq. 5)	$f_{cd}(\varvec{\xi }_n)$	Avoid overlapping
	Target reachability (Eq. 6)	$f_{rea}(\varvec{\xi }_n)$	Check task feasibility
	Layout compactness (Eq. 7)	$f_{c}(\varvec{\xi }_1,..., \varvec{\xi }_N)$	Reduce material handling cost
	Production time (Eq. 8)	$f_{t}(\varvec{\xi }_1,..., \varvec{\xi }_N)$	Improve production efficiency

**Robot singularity**: During the production process, if the fifth joint is zero, there will be wrist singularity issue for robots as given in Fig. 7, which makes the robot difficult to manipulate. For improving manipulability, singularity is also being detected and avoided in our work. Given $\varvec{A} = \varvec{JJ^T}$, where $\varvec{J}$ is the Jacobian matrix, the manipulability $\mu _m (\varvec{A})$ of the robot can be defined as

$$\begin{aligned} \mu _m (\varvec{A}) = \frac{\sqrt{\lambda _{max}(\varvec{A})}}{\sqrt{\lambda _{min}(\varvec{A})}}, \end{aligned}$$

with $\lambda _{max}$ and $\lambda _{min}$ the maximum and minimum eigenvalues of $\varvec{A}$, which is also known as the longest and shortest axes of the manipulability ellipsoid. Consequently, the fitness function can be given as

4 $$\begin{aligned} f_{man}(\varvec{\xi }_n) = {\left\{ \begin{array}{ll} 0 &{} \text {if}\ \mu _m (\varvec{A}) < \mu _{0} \\ \mu _0 &{} \text {if}\ \mu _m (\varvec{A}) \ge \mu _{0}. \end{array}\right. } \end{aligned}$$

where $\mu _0$ is the threshold of the robot manipulability.

The self-interest behaviours are summarised in Table 4, along with the objective explanation of each fitness function.

#### Cooperative interaction

Besides self-interest interaction, cooperation among different agents is also commonly in multi-agent reconfigurable layout optimisation. In the following, we introduce a group of fitness function for cooperative interaction behaviour among various agents.

**Fig. 8:**
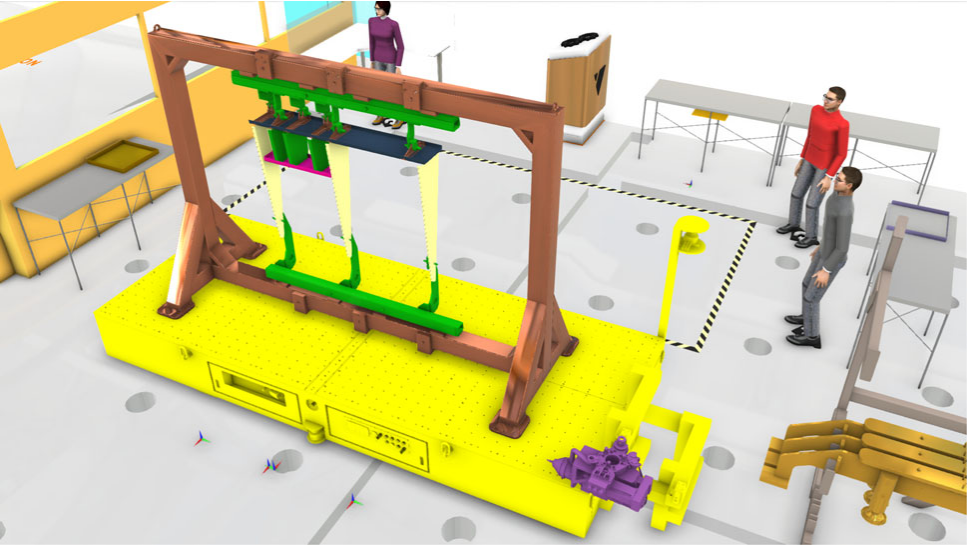
Collision detection. The collision among different agents is detected during the implementation of the production processes

**Collision detection**: Collision detection among agents is another key factor in layout design. In Fig. 8, the AGV has unexpected collision with the end-effector stand as shown in yellow. Based on the digital twin model, collision should be detected during the entire production process. Given the volumes of the cooperative agents, collision detection can be defined as

$$\begin{aligned} V_d = \sum _{n_d} V_m \cap V_n, \end{aligned}$$

with $V_m$ the checked agent and $V_n$ the *n*-th agent that cooperates with *m*-th agent during the production process. $n_d$ defines the numbers of collision detection. If the collision set $V_d$ is null, then *m*-th agent has no collision with the others. Hence the fitness function of collision detection function can be defined as

5 $$\begin{aligned} f_{cd}(\varvec{\xi }_n) = {\left\{ \begin{array}{ll} 0 &{} \text {if}\ V_d = \emptyset \\ V_0 &{} \text {otherwise}. \end{array}\right. } \end{aligned}$$

where $V_0$ is a constant value which is used to penalise the collision.

**Fig. 9:**
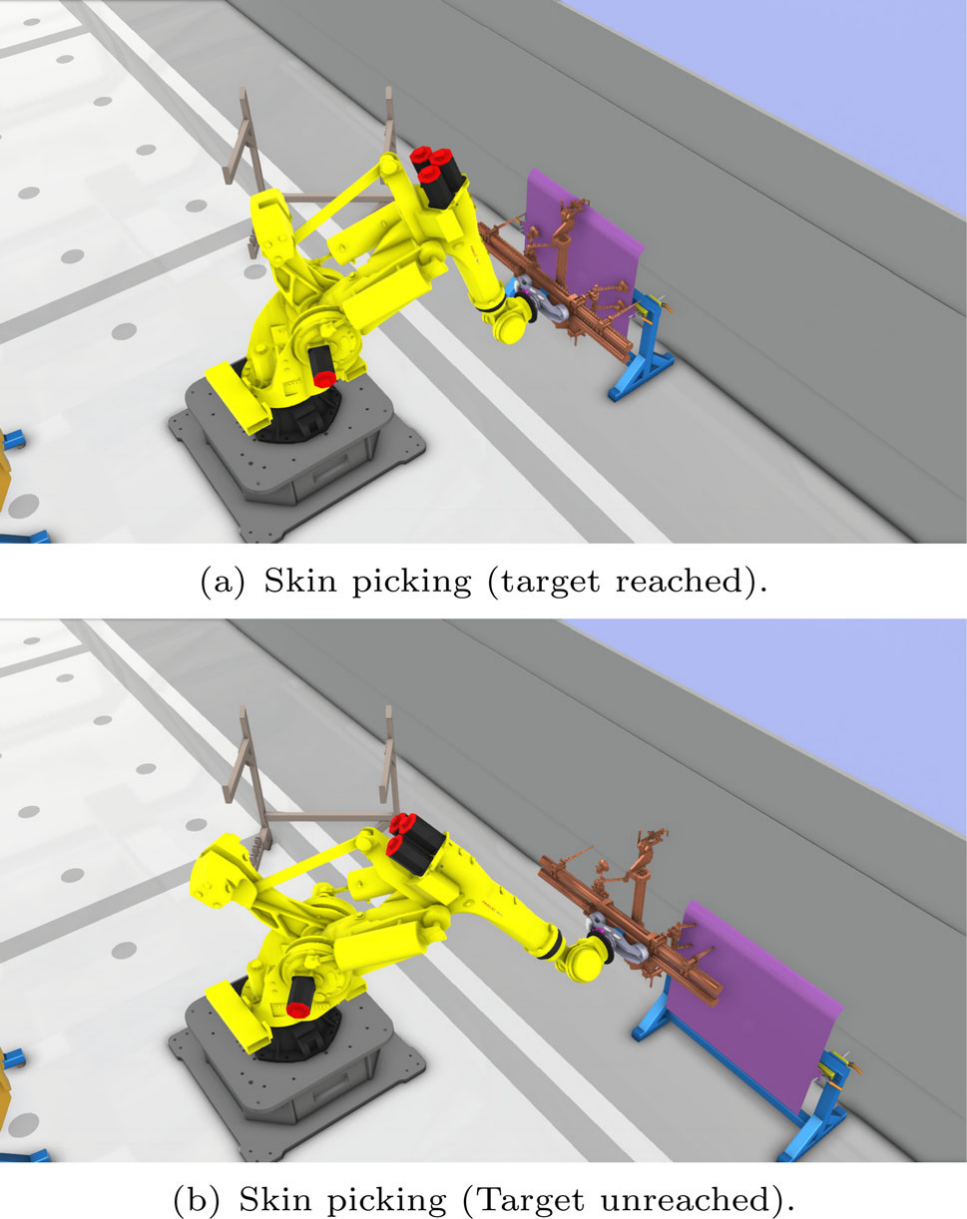
Target reachability issues triggered by relocation. As in **a**, the FANUC robot can pick up the skin within the workspace. However, in **b**, after relocation, the skin picking point is out of the robot workspace

**Fig. 10:**
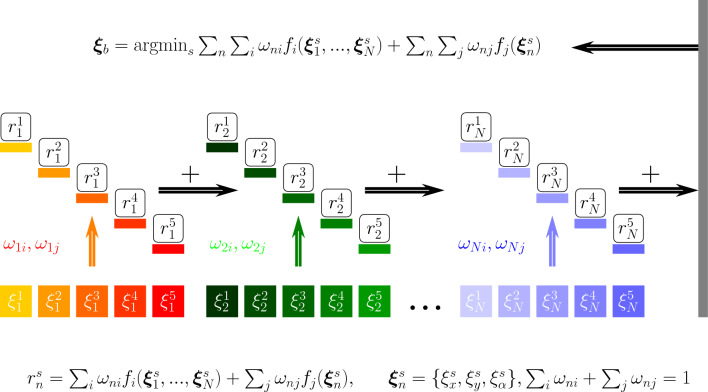
Cooperative swarm learning framework. The Cooperative swarm learning framework aims to allocate various agents $\varvec{\xi }_n^s$ with different fitness functions ($f_i, f_j$) and weights ($\omega _i, \omega _j$) into an overall learning scheme. Each agent explores the surrounding environment in the work cell separately and contributes to the whole learning reward cooperatively. The reward of each agent particle is defined as $r_n^s$ with *n* and *s* the agent label and particle label respectively

**Target reachability**: If the robot cannot reach the predefined target after relocation as shown in Fig. 9, the picking task will be uncompleted. For a dual agent task, the reachability check for a robot agent can be obtained by breaking the series chain of the robot into two parts, series chain $h(q_1,..., q_k)$ and series chain $g(q_{k+1},..., q_J)$, $1< k < J$, and hence check if there is a conjunction between two series sets.

$$\begin{aligned} \Lambda _{r} = h(q_1, ..., q_k)\cap g(q_{k+1}, ..., q_J) \end{aligned}$$

Therefore, the fitness function of the target reachability can be given as

6 $$\begin{aligned} f_{rea}(\varvec{\xi }_n) = {\left\{ \begin{array}{ll} 0 &{} \text {if}\ \Lambda _r \ne \emptyset \\ \sum _{n_\Lambda } \Lambda _0 &{} \text {otherwise}. \end{array}\right. } \end{aligned}$$

If the region $\Lambda _{r}$ is empty, then the target cannot be reached and the penalty constant $\Lambda _0$ will be accumulated. Otherwise, the target is within reach.

**Layout compactness**: The compactness of the system layout is equally important since it is directly related to capital cost, space and production cost. The fitness function of the layout compactness is expressed as

7 $$\begin{aligned} f_{c} = \sum _n \sum _m \Vert \varvec{\xi }_n - \varvec{\xi }_m \Vert ^2 \end{aligned}$$

where $\varvec{\xi }_n$ and $\varvec{\xi }_m$ are the positions of two agents, respectively.

**Production time**: The production time $f_{t}$ is also penalised in the framework developed, as it is the key performance indicator for the effectiveness of the overall layout optimisation result.

8 $$\begin{aligned} f_{t} = \mathop {\mathrm {arg\,max}}\limits \sum _{n_t} \Delta t_r^s \end{aligned}$$

where *s* is the product label and *r* is the process label. $n_t$ defines the number of sequential processes. Given that a manufacturing system may produce several different products and processes in parallel, only the maximum production time is taken into consideration in this work.

Similarly, the cooperation behaviours are concluded in Table 4, along with the objective explanation of various fitness functions.

## Cooperative swarm optimisation

After establishing the multi-agent RMS, this section focuses on exploring the optimal layout solution for the system. Firstly, the cooperative swarm learning framework regarding multi-objective fitness functions is proposed in Cooperative interaction. Secondly, the digital twin learning environment is introduced in Digital-twin based learning environment, along with the communication among various agents in Visual Components and TWINCAT.

### Cooperative swarm learning framework

Based on the self-interest and cooperation behaviours, the layout optimisation for robotic assembly cells is a multi-objective problem. As shown in Table 4, all fitness functions correlate to a unique position of a single agent or unique positions of several agents, which means the fitness functions are injective.

As shown in Figure 10, the layout optimisation problem can be mathematically defined as:

9 $$\begin{aligned} \begin{aligned} \min _{\varvec{\xi }_1, ..., \varvec{\xi }_N} \quad&\sum _i \omega _i f_i(\varvec{\xi }_1, ..., \varvec{\xi }_N) + \sum _j \omega _j \sum _n f_{j}(\varvec{\xi }_n) \\&i = \{ c, t\}, j = \{ inv, man, rea, cd, arr \} \\ \text {s.t.} \quad&\varvec{\xi }_n \in [\varvec{\xi }_n^l, \varvec{\xi }_n^u]\\&h(\varvec{\xi }_1, ..., \varvec{\xi }_N) = 0 \\\&\sum _i \omega _i + \sum _j \omega _j = 1 \\ \end{aligned} \end{aligned}$$

with $\omega _i$ and $\omega _j$ being the weight parameters for adjusting the multi-objective optimisation and superscript *l* and *u* being the lower bound and upper bound according to the layout area. In addition, $h(\varvec{\xi }_1,..., \varvec{\xi }_N)$ is a constraint equation used to further limit the searching areas in the manufacturing layout. $f_i$ represents the fitness function detailed in Agent state, action and communication and Cooperative interaction and summarised in Table 4.

**Fig. 11:**
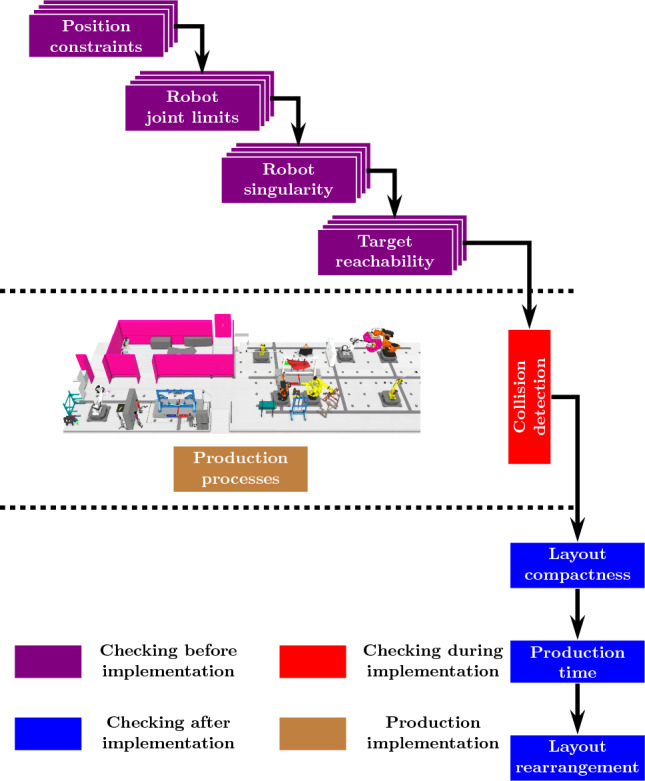
Calculating procedures of different fitness functions. Given that two types of interactions introduced in this work, the self-interest fitness functions are checked before the implementation of the production processes. However, since there might be clash during the implementation, the collision is detected throughout the whole implementation. Finally, the layout compactness is calculated after the implementation, along with production time and layout arrangement cost

During the layout optimisation, the fitness functions are calculated according to the flowchart given in Fig. 11. The fitness functions, robot joint limits $f_{inv}$, singularity $f_{man}$ and target reachability $f_{rea}$ are computed before the virtual production processes implementation. Then, the collision $f_{cd}$ among different agents is detected during the virtual production processes implementation. Finally, the other fitness functions are calculated after the implementation.

Regarding the population-based approach, the partial swarm optimisation and multi-agent system do share some similarities. Firstly, the agent in the layout could be seen as a particle. During the exploration of a particle in the problem space, the position of an agent is adjusted according to the penalty derived from the fitness functions. Secondly, both approaches requires cooperation among different components. The particle swarm optimisation updates the next-step position and velocity according to the evaluation of each particle exploration. While for the multi-agent system, it combines the self-interest and cooperative interactions to explore the surrounding environment.

Nevertheless, simple combination of particle swarm optimisation and multi-agent system is not applicable given the following reasons. Initially, not all the agents share the same fitness functions. For instance, the storage agent in the manufacturing layout would only require optimisation for its position to avoid collision and increase compactness. While for the manipulator, the joint limits, singularity and target reachability should be taken into consideration overall. Moreover, particle swarm optimisation explores the optimal solution or suboptimal solution in an overall environment towards a single objective or multiple objectives. On the other hand, layout optimisation of multi-agent system usually employs agents which are learning in different environments with non-identical multiple objectives.

**Fig. 12:**
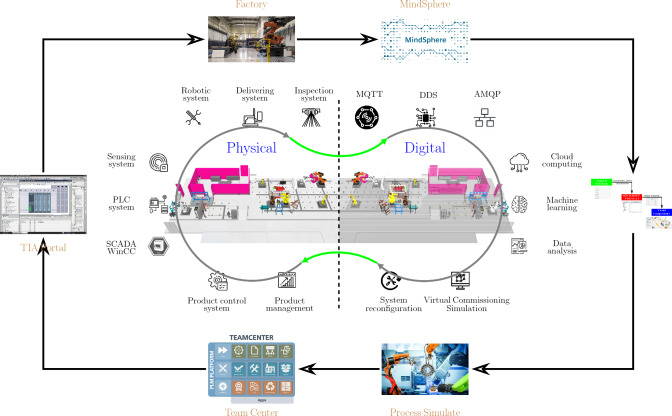
The position of our proposed dynamic layout optimisation framework in the entire Omnifactory system

Therefore, a cooperative swarm learning framework is introduced here for layout optimisation of the reconfigurable robotic assembly cell. In this learning framework, each agent learns two types of interactions, self-interest and cooperative as shown in Table 4. For layout optimisation, the position parameters $\varvec{\xi }_n^s = (\xi _x^s, \xi _y^s, \xi _{\alpha }^s)$ of the agent, including two translational and one rotational variables, are considered as the learning variables, with *s* being the particle index.

In the cooperative optimisation scheme, each agent equipped with *S* particle sets searches the surrounding and enrich the local view of the overall environment. During the exploration, different allocations of the particles generate different penalties derived from the fitness function described in Table 4. Meanwhile, the weight parameters are defined according to the functionality of the agent. For example, a robot agent would be analysed in terms of joint limits, singularity and target reachability. Therefore, the corresponding weight parameters are set to zero. Consequently, an extra agent label *n* to the weight parameters $\omega _{ni}, \omega _{nj}$ is added to customise the penalty for various agents.

**Fig. 13:**
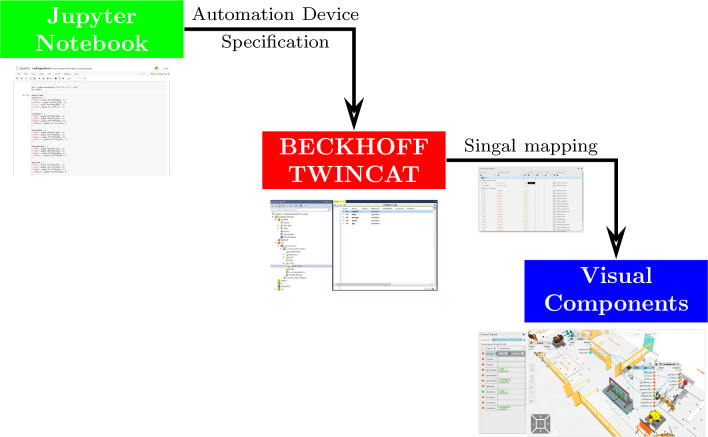
Digital twin learning environment.The digital twin models are established in Visual Components, where the communication among different components is event-triggered internally. Moreover, overall cooperative learning framework is embedded in Jupyter Notebook. Hence, the signals are connected externally through TWINCAT (Automation Device Specification, ADS)

During each learning episode, the reward of an agent with a particle *s* can be derived from the customised fitness functions as shown in Fig. 10.

10 $$\begin{aligned} r_{n}^{s} = \sum _i \omega _{ni} f_i(\varvec{\xi }_{1}^s, ..., \varvec{\xi }_{N}^s) + \sum _j \omega _{nj} \sum _n f_{j}(\xi _{n}^s) \end{aligned}$$

where $\varvec{\xi }_n^s = \{ \xi _x^s, \xi _s^y, \xi _{\alpha }^s \}$. The reward of all the agents with customised weighted fitness functions can be given as $r_s = \sum _n r_{n}^s$ for the current learning episode. Hence, the global best position $\varvec{\xi }_{n}^b$ is correlated to the minimum reward choosing from *S* explorations of the current episode. Then the next-step $t+1$ velocity and the position of a particle can be defined as

11 $$\begin{aligned} v_{nd}^s(t+1)&= v_{nd}^s(t) + c_1 u_1(t) [\xi _{nd}^{sb}(t) - \xi _{nd}^s(t)] \nonumber \\&+ c_2 u_2(t) [\xi _{nd}^{b}(t)- \xi _{nd}^s(t)] \nonumber \\ x_{nd}^s(t+1)&= x_{nd}^s(t) + v_{nd}^s(t) \end{aligned}$$

with $c_1$ and $c_2$ being the positive acceleration constants, which are applied to improve the cognitive and social behaviours, respectively. $u_1(t)$ and $u_2(t)$ are random values derived from the uniform sampling.

**Fig. 14:**
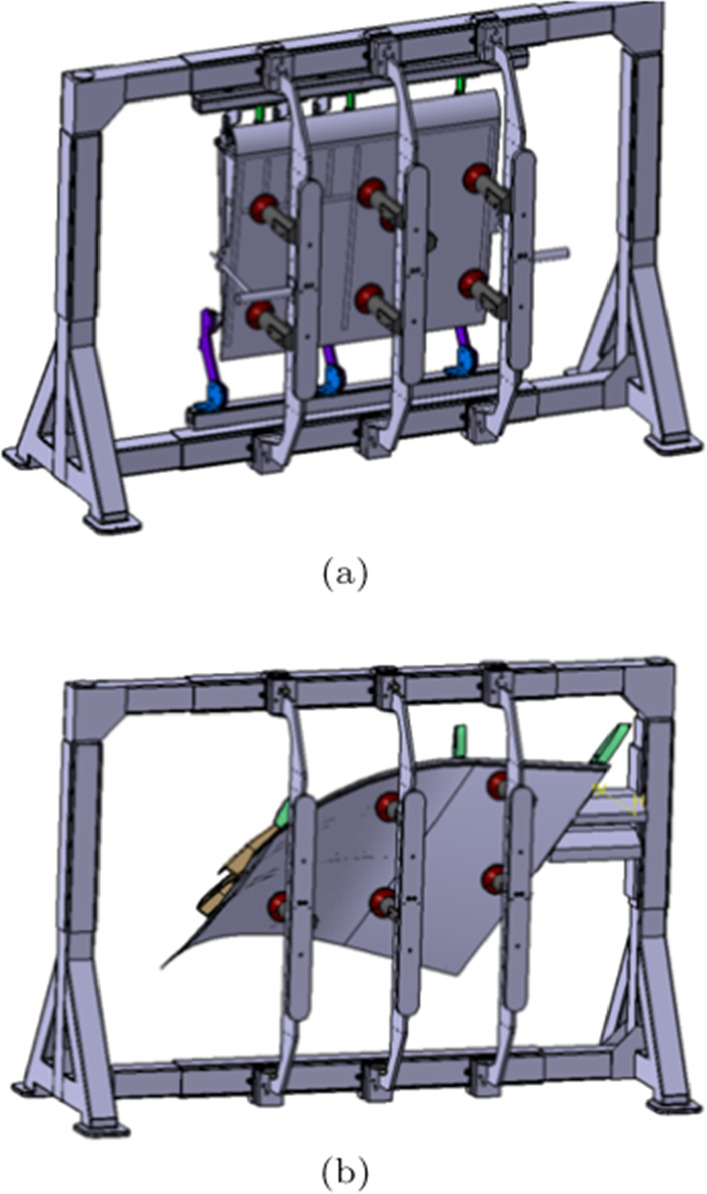
Frame for different product assembly. The designed frame aims to allocate different product assembly in a reconfigurable manner as shown in **a** and **b**. The vacuum cups located on the profile boards can be flexibly adjusted to hold different products

### Digital-twin based learning environment

A digital twin is the virtual representation of a manufacturing system. More specifically, it duplicates the exact physical devices and machines in the system layout. As presented in Fig. 12, both physical facility layout and its digital twin are established in the overall Omnifactory system. The physical side consists of manipulators, AGVs and inspection systems. Their data (configuration, task parameters, and real-time sensing information) are obtained through various communications such as MQTT, DDS, and AMQP into Siemens MindSphere. Then, based on the digital twin model established in VC, the layout is optimised given, task parameters, novel requirements and device configuration.

In addition, the optimisation result is further sent to Process Simulate for virtual commissioning and PLC code generation as indicated in Fig. 12. In Siemens Team Centre, the product management and production control system are implemented. Finally, the logic control, sensing system and HMI are designed in the TIA Portal for controlling physical side in Omnifactory.

Consequently, embedded into the Omnifactory system as shown Fig. 12, our proposed multi-agent dynamic layout optimisation can be divided into three levels as illustrated in Fig. 13.

At the bottom level, Visual Components (VC) simulation software is employed to establish the digital twin of the manufacturing system. More specifically, the behaviour of each component can be programmed in Python, as well as the management of their allocations. In addition, the cooperative learning framework is in the top level, along with data collection and data analysis. The learning framework is programmed and edited via Jupyter Notebook.

At the middle level, the connection for the learning environment is guaranteed with BECKHOFF TWINCAT. Based on the Automation Device Specification (ADS) protocol, the data, which contains the behaviour information of each component or agent, is communicated among three levels. The advantage of using TIA-portal is its capability to effectively connect to the physical PLC system and transfer the learning result in a batch form. The behaviour data of each agent is collected after each episode and the parameter of the cooperative framework is updated after each exploration, which contains a number of episodes.

## Evaluation

In this section, the proposed cooperative learning framework is evaluated with two use cases. The first use case is derived from the first station in Omnifactory, which is a reconfigurable robotic assemble cell with dynamic layout requirements, such as low rearrangement cost and material handling cost. As this system has been optimised with our proposed learning framework and successfully applied in the real world, the learning result will be detailed in Frame assembly work cell, along with the assembly implementation.

In addition, a more comprehensive analysis of the proposed layout learning framework is performed for the second station in Omnifactory as given in Drilling work cell. The second experiment aims to provide a thorough analysis by prioritising different learning objectives.

### Frame assembly work cell

As presented in Fig. 14, the work cell aims to assemble a family of small-box products. With adjustment of the vacuum cups and locating pins located on the profile boards as given in Fig. 14a and b, the frame can support the assembly of different products. The design objective for this work cell is to achieve low cost material handling (the frame will be located on an AGV in the next work package) and assembly system rearrangement. In addition, the assembly processes should be feasible and any damage or collision should be avoided.

In terms of resources, there are two KUKA KR270 robots, a profile board storage rack, a end effector tool stand and a frame allocated in this work cell. The digital twin of the whole work cell is established in Visual Components as detailed in Digital-twin based learning environment. According to the learning environment detailed in Fig. 13, the digital model is established in Visual Components and the cooperative learning framework is programmed via Jupyter notebook. Finally, the connection between the high-level learning framework and low-level digital twin is achieved with BECKHOFF TWINCAT3 (APS, pyads Python package).

**Fig. 15:**
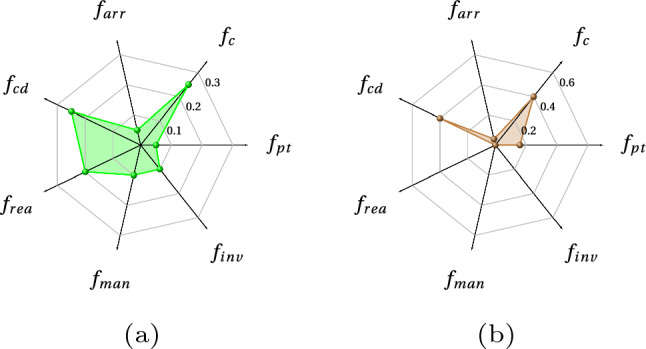
Kiviat chart of the weight parameters for agents. **a** Weight parameters for the Kuka robot. **b** Weight parameters for the tool stand, storage, and frame

**Fig. 16:**
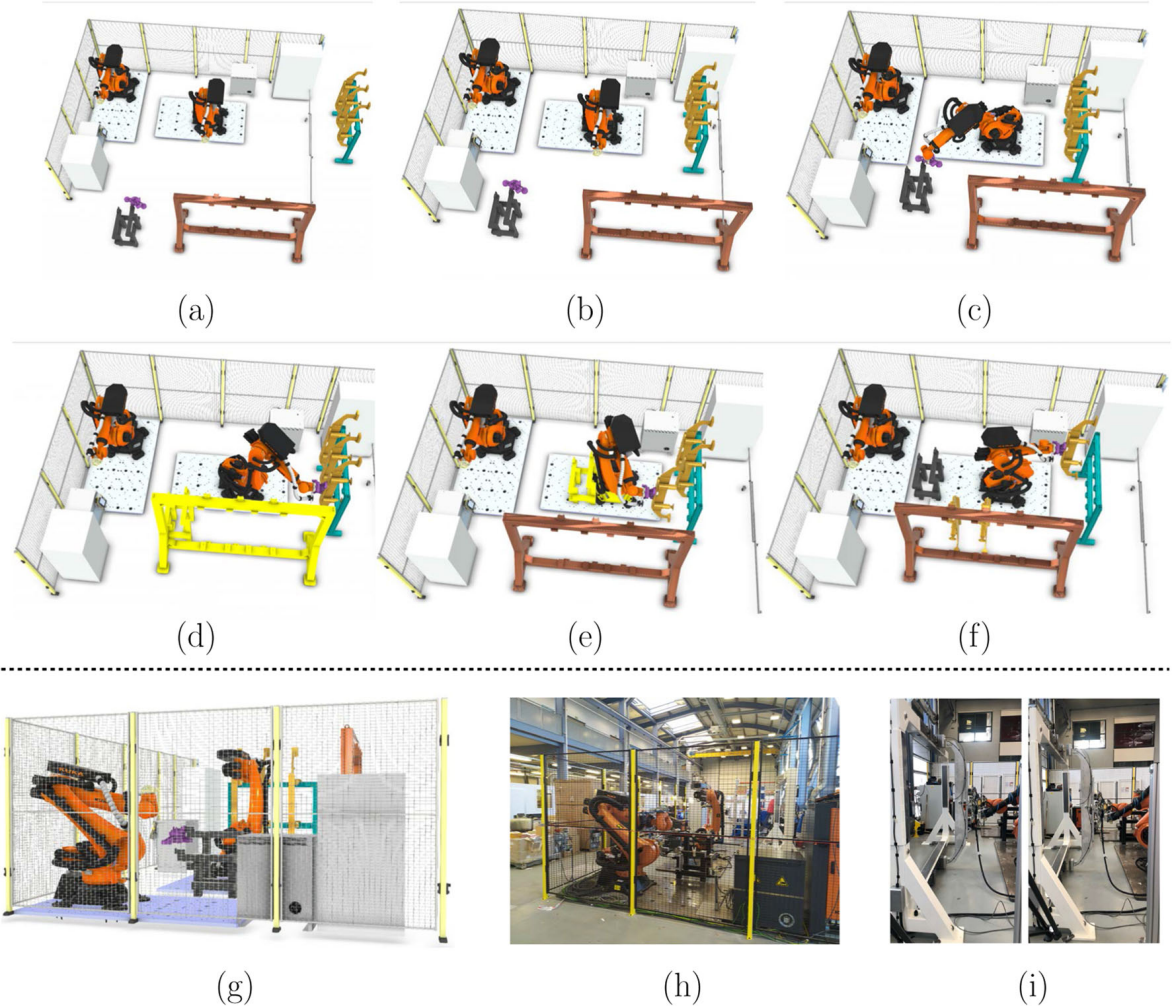
Experimental learning result and real-world assembly implementation. The snapshots of the cooperative learning for the assembly work cell are presented from **a** to **f**. In addition, the learned result (digital twin of the assembly work cell) and its physical side are given in **g** and **h**, respectively. Finally, the real-world assembly is carried out as indicated in **i**

**Fig. 17:**
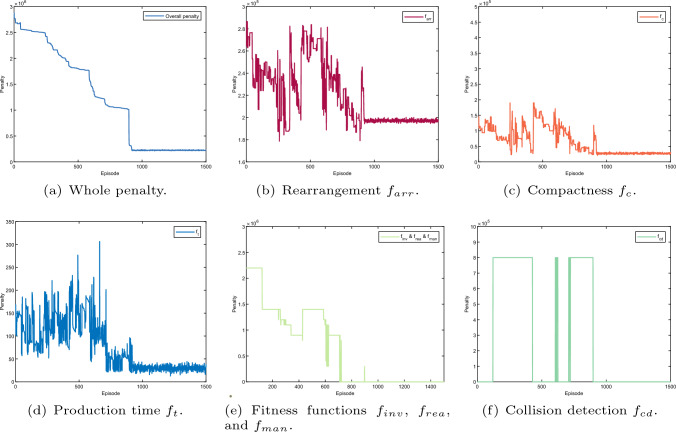
Layout optimisation result (all four agents). The sum of the rearrangement cost is given in Fig. 17b, including the rearrangement cost of each agent as presented in Figs. 18c, 19c, 20c, and 21c. The sum of the compactness cost is shown in Fig. 17c, consisting of the compactness cost of each agent as given in Figs. 18b, 19b, 20b, and 21b. The learning cost of the fitness functions, i.e., robot joint limits, singularity and target reachability are given in Fig. 17e, which is only addressed by KUKA agent. Regarding the learning cost of the production time fitness function and the collision detection fitness function are given in Figs. 17d and 17f, which apply to all four agents

**Fig. 18:**
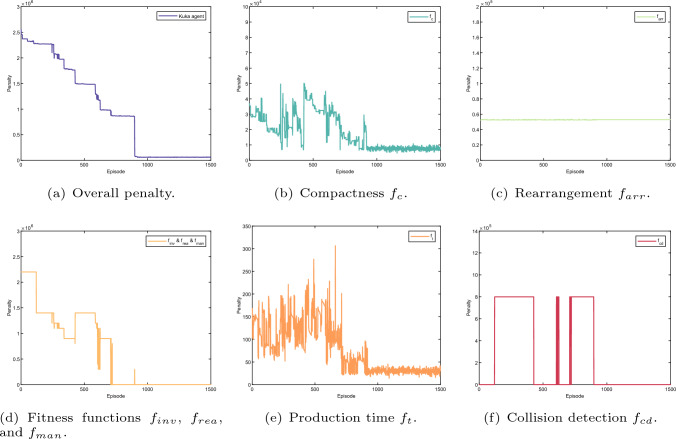
Layout optimisation result (KUKA agent). The overall penalty regarding learning episode of the KUKA agent as given in **a** is the sum of rhe penalty for compactness, rearrangement cost, robot configuration, production time and collision detection as presented in **b**–**f**, accordingly. Note that, the compactness penalty, rearrangement penalty and robot configuration penalty belongs to KUKA agent itself. However, As the production time fitness function and collision detection are designed for all four agents, the production time and collision detection are identical for all four agents

**Fig. 19:**
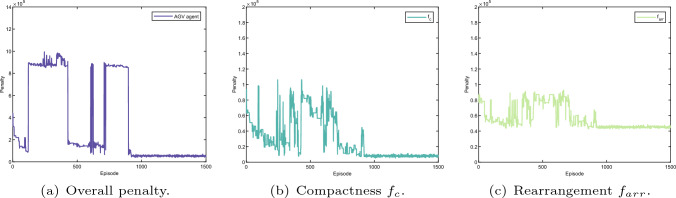
Layout optimisation result (frame agent). The whole penalty regarding learning episode of the frame agent as shown in **a** consists of the penalty for compactness, rearrangement, production time and collision detection as indicated in **b**, **c**, Figs. 18e, and f, respectively

**Fig. 20:**
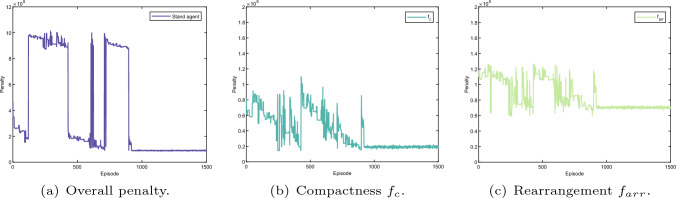
Layout optimisation result (stand agent). The whole penalty regarding learning episode of the stand agent as shown in **a** consists of the penalty for compactness, rearrangement, production time and collision detection as indicated in **b**, **c**, Fig. 18e, and f, respectively

**Fig. 21:**
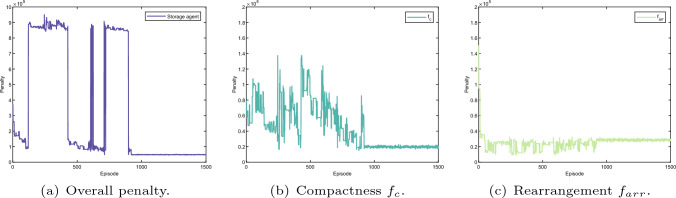
Layout optimisation result (storage agent). The whole penalty regarding learning episode of the storage agent as shown in **a** consists of the penalty for compactness, rearrangement, production time and collision detection as indicated in **b**, **c**, **e**, and **f**, respectively

Before the layout optimisation starts, weight parameters for each agent are set as shown in the Kiviat chart in Fig. 15. The weight parameters for the Kuka robot is indicated in Fig. 15a. The scenarios for the other three agents are set identically in Fig. 15b.

According to the project requirement, the layout of the whole work cell should be allocated in a compact space (fitness function $f_c$). Therefore, the weight parameter for compactness is set to be 0.5, which is the largest amongst all weight parameters. However, the target reachability, robot joint limits and singularity are also essential as they can decide the feasibility of the whole assembly process. Therefore these penalties ($f_{rea}, f_{man}, f_{inv}$) are included in the robot agent as presented in Fig. 15a. The other demand such as production time $f_t$ and rearrangement cost $f_{arr}$ are also taken into consideration.

Different devices (agents) are located in a reasonable space within the designated work cell, and the initial layout is presented in Fig. 16a. Initially, each component is relatively far away from each other. In the following multi-agent cooperative swarm learning, agents in the cell start approaching each other, as given in Fig. 16b, c, d, and e. However, at one point, the cell is so compact that collisions among agents are detected as illustrated in Fig. 16d and e. As given in Fig. 16e, the tool stand agent has clashed with the manipulator. However, these collisions can be penalised significantly with the collision detection fitness function.

Therefore, the framework enables learning from the rewards and penalties, and the agents would relocate in further learning processes. The final optimised layout is presented in Fig. 16f. In addition, during the real-time implementation, the updated digital twin as shown in Fig. 16g provides lifecycle support for the physical side as given in Fig. 16h. Correspondingly, the process of the robot mounting the profile board onto the frame is presented in Fig. 16i.

The learning results for all agents during the optimisation are given in Figs. 18, 19, 20 and 21. The overall penalty of KUKA robot as shown in Fig. 18a consists of five fitness functions including compactness (Fig. 18b), rearrangement (Fig. 18c), robot reconfiguration (Fig. 18d), production time (Fig. 18e) and collision detection (Fig. 18f). In contrast, the frame agent contains four fitness functions such as compactness (Fig. 19b), rearrangement (Fig. 19c), production time (Fig. 18e) and collision detection (Fig. 18f). Similarly, the penalty of the stand agent and the storage agent also has four fitness function as given in Figs. 21a and 20a, accordingly.

Note that, as the fitness functions of collision detection and production time are penalised for all four agents, the learning results of collision detection (Fig. 18e) and production time (Fig. 18f) apply to all four agents. In addition, without loss of generality, dynamic layout of the robotic assembly cell is optimised relatively to the KUKA robot. Hence, the arrangement cost of the KUKA robot as given in Fig. 18c remains constant during the learning process.

Additionally, penalties for all the fitness functions are presented in Fig. 17b–f. The dynamic layout optimisation is converged after nearly 1000 episodes. Although the overall penalty of the work cell gradually decreases during the learning, the penalties for four agents do not have the same trend as presented in Figs. 18, 19, 20 and 21. Initially, the layout is loose, there is no collision among different agents given in Fig. 17c and f. However, the sum of singularity, reachability and joint limits indicate that the assembly targets are actually out of the manipulator’s workspace as shown in Fig. 17e.

At the episode around 200, as the layout shrinks, penalties of the manipulator ($f_{inv}, f_{rea}, f_{man}$) decrease as indicated in Fig. 17e. Nevertheless, collision is detected at the same learning period, which indicates that the layout is too compact. The same penalties from the agents are also shown in Figs. 18f,  19a, 20a, and 21a. Note that, as long as a clash is detected, all the agents are heavily penalised in the optimisation framework.

**Fig. 22:**
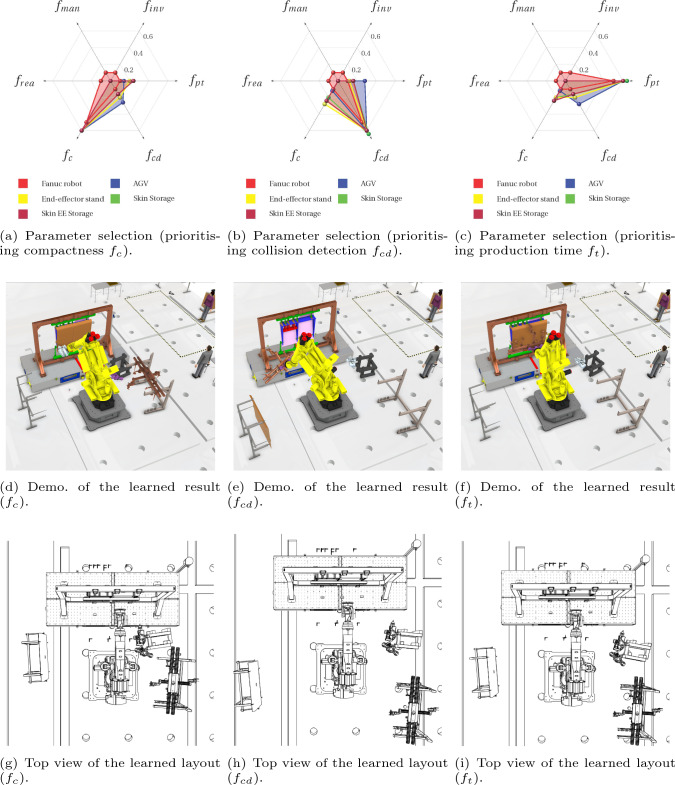
The parameter selections, the last-step demonstrations and the top views of the learned layouts of the second experiment. The corresponding optimised result prior to compact fitness function $f_c$ is given in **a**, **d** and **g**. The corresponding optimised result prior to collision detection fitness function $f_{cd}$ is illustrated in **b**, **e** and h. The corresponding optimised result prior to production time fitness function $f_{t}$ is illustrated in (**c**), (**f**) and (**i**)

The most interesting part is from around episode 450 to episode 550. The layout is enlarged by a small scale, and no collision was triggered. Although the penalties for the Kuka robot ($f_{inv}, f_{rea}, f_{man}$) are shown at a large value, the overall penalty is decreasing as shown in Fig. 17e. This is because in the multi-agent system, the agents learn from collective reward in each exploration. Although not all of the agents show the same trend of convergence (as presented in Figs. 18, 19, 20, and 21), the overall penalty is decreasing during the learning. Therefore, after nearly 950 episodes, the penalties for every fitness function converge (as given in Fig. 17b, c, d, e and f) and the layout is at an optimised configuration after nearly 1000 episodes.

In summary, compared with the initial facility layout, the rearrangement cost is reduced by 33.4$\%$. The overall layout compactness among different agents is improved for 3.8 times and the reduction of the production time is at 2.3 times. Moreover, after the layout optimisation, there is no collision detected during the entire process implementation and there is no joint limit, reachability and singularity issues at any assembly points.

### Drilling work cell

In the second scenario, the proposed cooperative learning framework is exploited for its ability to select weight parameters for different fitness functions. The layout reconfiguration task is to optimise the work cell with one Fanuc robot. More specifically, the Fanuc robot assembles the skin to the generic hinged product with the skin end-effector and then, drill on the skin with the drill end-effector.

Since this case study is used to demonstrate and discuss the selection of the weight parameters, we design three parameter selection scenarios as shown in Fig. 22a, b and c. As presented in Fig. 22a, the layout compactness is set to be the largest weight parameter for all five learning agents in the work cell, namely Fanuc robot, AGV, end-effector stand, skin storage and skin end-effector storage. In this way, the optimisation framework is prone to choose a more compact layout. Similarly, in Fig. 22b, collision detection is set to be the largest weight parameter in order to prioritise collision-free layout reconfiguration. In Fig. 22c, the consideration priority is the production time.

The experimental setting is the same as the first experiment, as well as the learning environment. The particle quantity for each agent is set to be five. The optimal layouts are illustrated in Fig. 22d, e and f, respectively. For further clarity, top views for the optimised layouts are shown in Fig. 22g, h and i.

As presented in Fig. 22g, the reconfigured layout is the most compact compared with the other two given in Fig. 22h and i. This is because of the larger penalty relating to the compactness fitness function as shown in Fig. 22a. In addition, the optimal layout solution prioritising the production time fitness function $f_{t}$ is illustrated in Fig. 22f and i. Furthermore, when comparing layouts from Fig. 22g and i, it shows that a more compact layout does not always lead to less production time. This is because the manipulator point-to-point motion is not simply planned by the straight line connecting these points (Laumond et al., 1998).

**Fig. 23:**
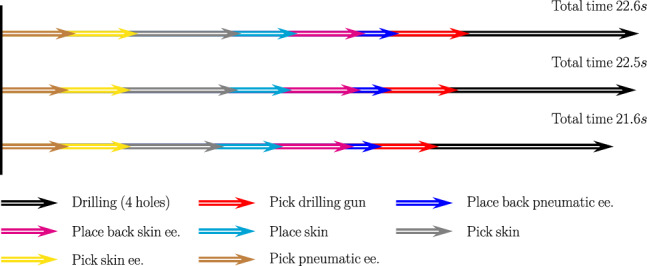
Comparison of the elapsed time of the three optimisation schemes. For the three optimisation schemes, the elapsed time for each production process are presented in the above figure. The total time is listed at the end of each optimisation scheme

In order to provide a more comprehensive explanation, the elapsed time for each process is listed in Fig. 23. It shows that the compact layout ($f_c$) spends more time on picking up the pneumatic and drilling end-effectors, as the robot takes time to accelerate and decelerate. For the $f_{cd}$ case, the skin picking up process takes longer than the other two layout configurations. However, after the layout optimisation prioritising production time function, the total time reduces from 22.5*s* to 21.6*s*, around 4$\%$ improvement.Although, the improvement is only related to one work cell. When it comes to multiple work cells with repetitive operations, the improvement will be more significant.

In terms of the optimised layout prioritising collision detection $f_{cd}$, the components are located further away from each other in the work cell in Fig. 22h in contrast to the layout in Fig. 22g and i. However, the available workspace for a manufacturing system is often limited. The optimised layout can only be a viable solution when the workspace is big enough to accommodate it.

## Discussion

Since the proposed layout optimisation framework focuses on two successive levels, namely machine level and station level, with all the production manufacturing processes predefined in the digital-twin environment at the machine level. The robot joint limits, manipulation, and singularity are taken into consideration, they require large penalties to guarantee the success of motion planning and layout optimisation, which requires experience of choosing proper weight parameters. We are currently testing the dynamic layout optimisation issues in a hierarchical manner. Moreover, the machine level optimisation is implemented after the optimal solution at the station level layout. This could avoid manual weight parameter assignment and to some extent reduce conflicting issues amongst multiple objectives.

In addition, although the proposed framework supports random initial positioning, the learning timing and optimisation efficiency is sensitive to the initial layout, and the framework is much more efficient if a reasonable initial layout, or for a selected area, is given.

Due to changes in production requirements, expansion regarding new products and addressing deficiencies in the current facility reconfiguration is extremely necessary. Since the robotic cell is designed for reconfiguration, maximising asset reuse and promoting sustainable production, asset relocation and commissioning become recurring costs compared to traditional production systems. The proposed framework optimised facility layout considering objectives for reducing reconfiguration cost. Resiliency can only be achieved if the reconfiguration is timely, and this would rely on digital tools such as the framework proposed. The proposed layout optimisation framework focuses on the quick adaptation to unexpected production demands and increases resiliency by allowing timely decisions of facility layout in response to market change. Therefore by incorporating the proposed optimisation framework, the facility layout can contribute to long-term success of broader production and maintain both resiliency and sustainability.

The proposed framework was inspired initially as a layout planning tool to support the reconfigurable shop floor and accelerate the reconfiguration process by detailed simulation, comparison and decision-making in the early development stages. In the case where a business do not have the rapid physical-reconfigurability, they would still benefit from the digital-twin enabled information for decision-making. The framework can still provide optimal layout information regardless, for initial factory setup or reconfiguration. Based on the different level of physical reconfigurability, weight parameters for rearrangement cost will be different and assigned to limit the physical movement required. Therefore, we believe this framework could also be extended to manufacturing SMEs, as the penalty functions are similar and the theory behind this is identical.

## Conclusion

In this paper, a novel cooperative swarm learning framework for layout optimisation of a multi-agent reconfigurable robotic assembly cell is proposed based on its digital twin. In the evaluation, two use cases are presented in order to demonstrate the application feasibility of the proposed framework.

Based on the digital twin, a layout optimisation process is implemented under a multi-agent system framework consisting of self-interest and cooperative fitness functions. During the exploration, weight parameters provide further options for layout optimisation prioritising different objectives. The whole framework is initially verified with a reconfigurable assembly cell. The optimised reconfigured layout is successfully applied for ongoing projects at the University of Nottingham. According the layout optimisation result, the rearrangement cost is decreased by 33.4 $\%$. The layout compactness and the production time are improved for 3.8 times and 2.3 times respectively. During the overall assembly process implementation, there is no collision detected or any robot configuration issues such as joint limits, reachability and singularity at assembly points. Then, selection of the weight parameters is further discussed in the second use case. Three options, such as prioritising production time, collision detection and compactness are discussed regarding the corresponding layout.

Although this paper aims to address dynamic facility layout issues for a single robotic assembly cell, it can be extended to multiple facility layout problems. Moreover, the dynamic layout optimisation is investigated at both machine level and station level in this paper. For multiple facility layout optimisation, it will be at factory level. However, as the fitness functions are only dependent on facility positions, the proposed framework can run in parallel for each work cell separately.

In terms of product family, small box product family is within consideration, which includes winglets, rudders and elevators. Given that the assembly of aerostructures still requires high flexiblity, a manual assembly cell is also included in Omnifactory. To enable better awareness of the digital system, Siemens real-time tracking system is applied to monitor the usage of different tools, such as drilling and sealing guns.

Finally, the digital twin in this work is developed for dynamic layout optimisation as given in Fig. 12. Currently, the digital twin with the optimised layout result can be applied further to simulate PLC signals and generate off-line robot motion planning in Omnifactory. Given sensor information derived from MindSphere, the digital twin model can be viewed in real-time and provide specific factory information for the physical world.
